# Microwave Absorption of Crystalline Fe/MnO@C Nanocapsules Embedded in Amorphous Carbon

**DOI:** 10.1007/s40820-020-0388-4

**Published:** 2020-02-18

**Authors:** Gaihua He, Yuping Duan, Huifang Pang

**Affiliations:** Key Laboratory of Solidification Control and Digital Preparation Technology (Liaoning Province), School of Materials Science and Engineering, Dalian University of Technology, Dalian, 116085 People’s Republic of China

**Keywords:** Crystalline nanocapsule, Amorphous carbon, Core–shell structure, Interfacial polarization, Microwave absorption

## Abstract

Crystalline Fe/MnO@C core–shell nanocapsules inlaid in porous amorphous carbon matrix (FMCA) was synthesized successfully with a novel confinement strategy. The heterogeneous Fe/MnO nanocrystals are with approximate single-domain size which gives rise to natural resonance in 2–18 GHz. The addition of MnO_2_ confines degree of graphitization catalyzed by iron and contributes to the formation of amorphous carbon. The heterogeneous materials composed of crystalline–amorphous structures disperse evenly and its density is significantly reduced on account of porous properties. Meanwhile, adjustable dielectric loss is achieved by interrupting Fe core aggregation and stacking graphene conductive network. The dielectric loss synergistically with magnetic loss endows the FMCA enhanced absorption. The optimal reflection loss (RL) is up to − 45 dB, and the effective bandwidth (RL < − 10 dB) is 5.0 GHz with 2.0 mm thickness. The proposed confinement strategy not only lays the foundation for designing high-performance microwave absorber, but also offers a general duty synthesis method for heterogeneous crystalline–amorphous composites with tunable composition in other fields.

**Electronic supplementary material:**

The online version of this article (10.1007/s40820-020-0388-4) contains supplementary material, which is available to authorized users.

## Highlights

- The crystalline Fe/MnO@C core–shell nanocapsules embedded in porous amorphous carbon matrix (FMCA) was prepared by a novel confinement strategy of modified arc-discharge method.
- The heterogeneous crystalline–amorphous nanocrystals disperse evenly and exhibit improvement of static magnetization and excellent electromagnetic absorption properties.
- The adding MnO_2_ confines degree of graphitization and contributes to form amorphous carbon. Dielectric loss and microwave absorption are achieved adjustable.

**Electronic supplementary material:**

The online version of this article (10.1007/s40820-020-0388-4) contains supplementary material, which is available to authorized users.

## Graphical abstract

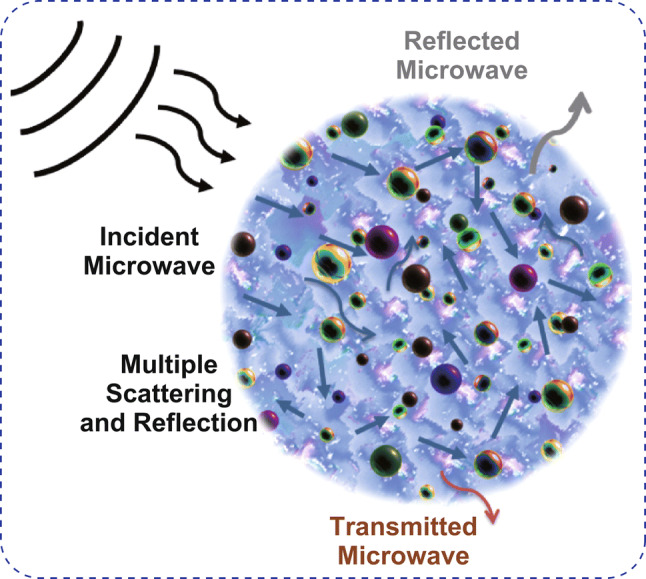

## Introduction

As science and technology develop dramatically fast, electromagnetic radiation pollution has become a growing problem due to the explosive growth in the application of electronic devices, such as radar systems, local area networks, telephones, and computers. Overexposure to electromagnetic wave can not only affect the functioning of electronic equipment but also be potentially detrimental to human being and raise severe problems concerning the field of military applications [1–3]. Therefore, it has spurred internationally unprecedented interest in exploring microwave absorbing materials, which converts electromagnetic wave into energy in other forms [4, 5]. For practical applications, desirable microwave absorbing materials are supposed to possess key features of high absorptive capacity, broad effective bandwidth, light weight, and low filler loading ratio [6–8]. Thus, it is urgent to develop materials with satisfaction of these conditions simultaneously.

Soft magnetic metal and alloys (i.e., Fe, Co, Ni, and their alloys) have been extensively applied for electromagnetic wave-absorbing materials because of their strong ferromagnetic properties, widely magnetic anisotropy, and popular domain wall displacement [9–11]. Nevertheless, the Snoek’s limit, poor impedance matching, large density, easy oxidation, and magnetic aggregation restrict their electromagnetic wave absorption property. In order to overcome these problems and further improve their microwave absorption properties, the magnetic materials are usually recombined with different dielectric materials, which is on base of the synergy of magnetic and dielectric mechanisms. Among plenty of dielectric loss medium, carbon materials are superior in the field of microwave absorption in virtue of their special merits, such as chemical stability, tunable physical and chemical properties, and various forms [7, 12–26]. Specially, massive efforts have been devoted continuously to developing absorbers, constituting carbon materials and magnetic metal iron composition recently. In past, the multicomponent hybrids Fe@C nanocapsules [27], Fe–C nanofibers [28], graphene-coated Fe nanoparticles [29], Fe@C microspheres [1], mesoporous Fe/C composition [30], C@Fe_2_O_3_/Fe_3_C/Fe–CNT nanoparticle decorated carbon nanotubes [31] display good electromagnetic absorption capabilities and wide effective frequency range to some extent.

Despite the promising progresses aforementioned, great problems remain to be resolved. At first, it is rather challenging and still imperative to tune the magnetic nanoparticles size and distribution by rational design and construction of architectures of microwave absorption materials to solve the problem of random distribution and serious aggregation. Second, carbon-magnetic composite possesses overhigh electrical conductivity owing to the laminated carbon structure, which results in the occurrence of significant skin effects under electromagnetic wave. Third, Fe_3_C is always inescapable accompanied by reaction. More importantly, the magnetic dilution will generate due to the introduction of nonmagnetic dielectric components.

It is inspired from researches to fabricate crystalline–amorphous composition comprised amorphous matrix and dispersed nanocrystalline [2, 32, 33], in which the unique construction conduces to the uniform dispersion of the magnetic nanoparticles, shows a high density interfaces and significant interfacial polarizations, benefiting for microwave attenuation. Hence, it is promising to find a simple method to construct heterogeneous material composed of crystalline–amorphous structure. One feasible approach is introduction of transition metal oxide MnO to construct heterogeneous crystalline–amorphous Fe–C-based composition with nanoscale architectures and further boost microwave absorption performance. The MnO in size of nanoscale contributes to produce more dipoles and dipolar relaxation [34]. MnO dispersed on the surface of graphene can also weaken the $\pi - \pi$ stacking interactions [35]. Otherwise, introducing MnO increases the heterostructure among MnO, Fe, and carbon, which can result in unpaired spins, form plentiful magnetic moments and further induce magnetic ordering in materials [36].

Herein, a kind of crystalline Fe/MnO@C core–shell nanocapsules inlaid in porous amorphous carbon was successfully prepared by arc-discharge method, which generates abundant interfaces because of graphitized graphene and amorphous carbon. The layer of graphene and size of dispersive Fe/MnO nanocrystal can be tuned by introduction content of MnO. And the magnetic dilution is compensated via enhanced surface anisotropy and resonance where Fe nanoparticles are confined about single-domain size. The novel configuration of Fe/MnO@C core–shell structure embedded in amorphous carbon not only suppresses the agglomeration of magnetic particles and consequent skin effect, but also provides good chemical homogeneity and sufficient interfaces between Fe/MnO nanoparticles and carbon. Besides, inescapable Fe_3_C accompanied by conventional reaction is suppressed in this method. On this basis, in corporation of magnetic metal with dielectric material, superb microwave absorption performance comes from efficient complementarities integrating permittivity with permeability. This study aims to develop convenient method to construct magnetic nanocrystals-carbon crystalline–amorphous structure high-performance microwave absorption. Moreover, it is to investigate the synergistic interaction between the magnetic nanocrystals and carbon matrix for practical applications in future.

## Experimental Procedures

### Synthesis

The porous amorphous carbon embedding crystalline Fe/MnO@C core–shell nanoparticles (FMCA) was prepared by an arc-discharged plasma technique. A compressed mixture of iron and manganese dioxide powders was attached to a water-cooled copper stage as the anode, and the carbon rod fixed on the opposite side served as the cathode. The chamber was evacuated to ~ 10^−3^ Pa, and then, the methane (CH_4_) was introduced to be 0.2 × 10^−3^ Pa as a gaseous source. The arc discharge was controlled simultaneously at ~ 30 V and ~ 90 A for 5–10 min by adjusting the distance between the two electrodes. The as-made FMCA nano-powders were collected after about 6 h and stored at ambient. The resultant FMCA were termed as FMCA-1, FMCA-2, FMCA-3, FMCA-4, FMCA-5, FMCA-6, and FMCA-7, corresponding to Fe mass ratios of 54%, 52%, 49%, 42%, 55%, 57%, and 60%, respectively.

### Characterization

The crystal structure of the samples was studied by X-ray powder diffraction (XRD, SHIMADZU, XRD-6000) with Cu Kα radiation, using an operation voltage and current of 40.0 kV and 30.0 mA, correspondingly. The scanning range was from the degree of 10° to 80° (2*θ*) and the scan speed was 4° min^A^. Transmission electron microscope (TEM) images were obtained by JEOLJMF-2100F field emission transmission electron microscope at an accelerating voltage of 200 kV. The chemical bonding state of the nanocomposites was analyzed by X-ray photoelectron spectroscopy (XPS). The magnetic hysteresis loop was determined by Lake Shore vibrating sample magnetometer (VSM) with a magnetic field range of 17 KOe.

### Electromagnetic Measurement

The relative complex permittivity and permeability versus frequency were obtained by coaxial reflection/transmission method using Agilent 8722ES vector network analyzer with the working frequency at 2–18 GHz. The cylindrical sample (with 3 mm in inner diameter, 7 mm in outer diameter and 2 mm in thickness) was fabricated by uniformly mixing paraffin matrix with 50 wt% absorbents. Then, the composite was pressed into cylindrical compacts. The transmission line theory was introduced to characterize the wave-absorbing properties, which is designated as reflection loss (RL).

## Results and Analysis

### Structure and Phase Characterization

**Fig. 1:**
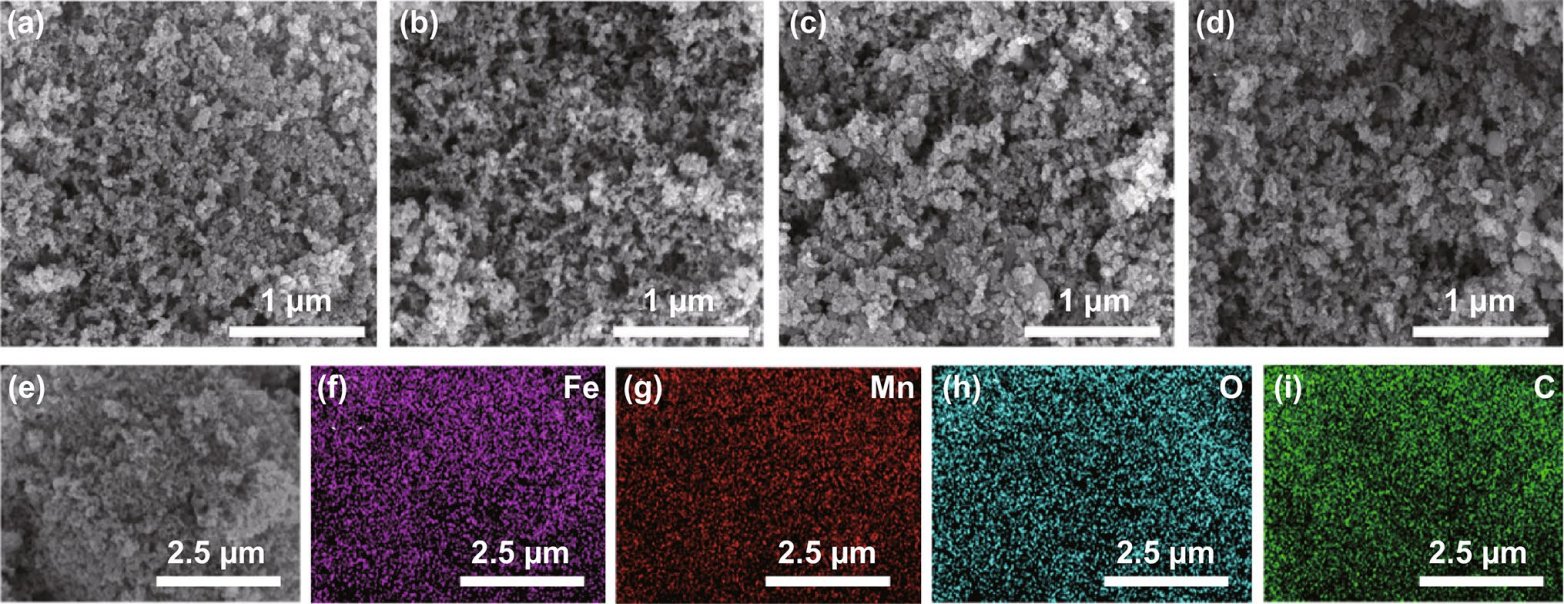
SEM images of different samples: **a** FMCA-1, **b** FMCA-2, **c** FMCA-3, and **d** FMCA-4. **e**–**i** Elemental mapping images of FMCA-1

The morphologies and structure are initially investigated by scanning electron microscope (SEM). All the samples display an aggregated yet microsphere-shaped morphology, as shown in Fig. 1a–d. The regular microspheres are uniform distribution. The element mapping reveals that Fe, Mn, O, and C elements are uniformly distributed in FMCA-1 (Fig. 1e–i).

**Fig. 2:**
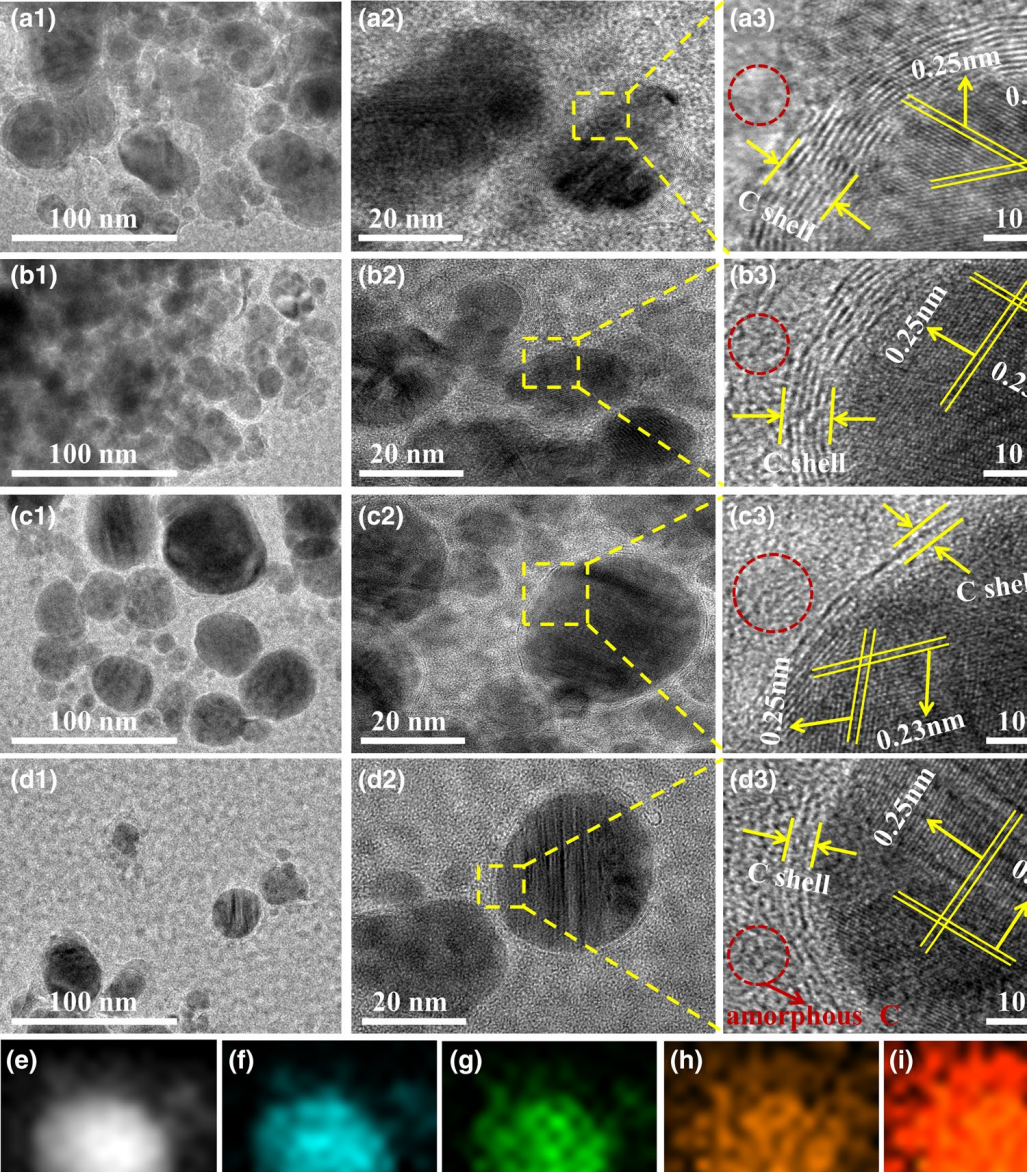
TEM and high resolution TEM images: **a1**–**a3** FMCA-1, **b1**–**b3** FMCA-2, **c1**–**c3** FMCA-3, and **d1**–**d3** FMCA-4. HAADF-STEM images of **e** single Fe/MnO nanocrystal and **f**–**i** elemental mapping images

Microstructure of the samples is further observed by TEM. Figure 2a_1_–d_1_ shows spherical Fe/MnO@C nanocapsules embedded in the amorphous carbon. Obviously, two kinds of carbon with different morphology of graphite carbon and amorphous carbon are formed by arc-discharge method. An enlargement of the nanocomposite in Fig. 2a_2_–d_2_ reveals that amorphous carbon wraps on Fe/MnO@C nanocapsules forming a double-shell microstructure. The HRTEM image indicates that Fe/MnO core is well coated by C shells. The embedded Fe/MnO@C nanocapsules are proved in very high degree of crystallinity. From Fig. 2a_3_–d_3_, the inter-plane distance between fringes of 0.25, 0.23, and 0.35 nm, correspond to the (110) crystal plane of metallic Fe, (200) plane of MnO, and (002) plane of graphitic carbon, respectively. Lots of distorted lattices are found on carbon-rich buffer layers. Some pronounced lattice defects, such as C-breakage and serious ripples, are present on the carbon layers, which mainly arise from the effect of energetic Ar^+^/H^+^ ions of plasma and the fast quenching by means of energy exchange during reaction process. Additionally, the addition of MnO_2_ confines growth of the Fe/MnO nanocrystal. The average size of Fe/MnO@C nanocapsules is approximately below 50 nm. Under the protection of carbon matrix, Fe/MnO nanocrystals show distinct morphology and fine dispersion without severe agglomeration.

The electron energy loss spectroscopy (EELS) elemental mapping of FMCA composition shows that Fe, Mn, and O elements completely overlap due to the diffusion of Fe and MnO during the sample preparation process (Fig. S1). The well distribution of Fe nanoparticles in Fe/MnO@C nanocapsules ascribe to the isolation effect of MnO and confine effect of graphite carbon and amorphous carbon matrix. Besides, the crystalline graphite thicknesses of samples FMCA-1, FMCA-2, FMCA-3, and FMCA-4 are 11, 7, 3, and 2–3 layers, respectively. The graphite layers decrease with the reduction of Fe content while increasing MnO content, which further manifests that some of the amorphous carbon could be transformed into graphite by Fe catalysis graphitization process. The different graphite thickness indicates different amount of hetero-interface in the composition. But it is hard to definite the number. The morphology with multiple interfaces is helpful for multiple interfacial depolarization, interfacial scatterings, and diffuse scattering of microwave. For comparison, Fe@C compositions are synthesized under the same condition without the addition of MnO_2_. In Fig. S2, the morphology of Fe@C compositions exhibits a reticulum-like porous structure, and Fe nanoparticles randomly distributes and encloses by the overlapped graphene nanosheets. High resolution TEM images in Fig. S2b illustrates the well-developed graphene layers with inter-plane spacing 0.36 nm wrapped on Fe nanoparticle, depicting the (110) crystal plane of metallic Fe phase with inter-plane spacing 0.25 nm. There is no observation of amorphous carbon in the Fe@C compositions. The highly graphitization degree may be harmful for the microwave absorption. By contrast, Fe facilitates the crystallization of carbon during the reaction, whereas MnO reduces the crystallization of carbon and contributes to the formation of amorphous carbon in the reaction. Moreover, the elemental mapping of a single Fe/MnO@C nanocapsules further manifests the distributions of Fe, Mn, O, and C element with uniformly and dispersity (Fig. 2e–i). The nanocapsules tightly encapsulated by carbon matrix would facilitate the transfer of charge carriers between Fe, MnO, and C, thus promoting excellent dielectric properties. Hence, this representative structure may contribute to electromagnetic attenuation.

**Fig. 3:**
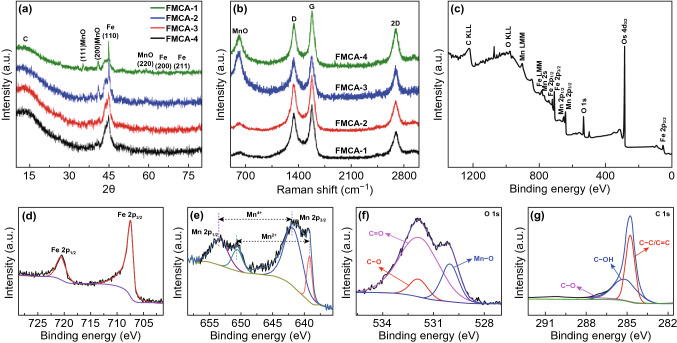
**a** XRD patterns of FMCA. **b** Raman spectrum of FMCA-3. **c** survey XPS of FMCA-3. **d** Fe 2*p*, **e** Mn 2*p*, **f** O 1s, and **g** C 1s spectra of FMCA-3

The crystalline structure of the synthetic nanocomposites is measured by XRD analysis (Fig. 3a). The diffraction peaks of 44.7°, 65.0°, and 82.3° are indexed to (110), (200), and (211) crystal planes of cubic Fe (JCPDS No. 06-0696). Three diffraction peaks appear at 35.0°, 40.7°, and 58.9° can be indexed to (111), (200), and (220) planes, respectively, of the cubic MnO (JCPDS No. 75-0626). The small hump is assigned to amorphous carbon, which indicates that the presence of MnO weaken the catalytic effect of iron species in the crystallization of carbon components [37]. The hierarchically porous structure of FMCA composition is further confirmed by nitrogen adsorption–desorption isotherms analysis. FMCA-3 reveals typical IV type curve, manifesting existence of amounts of mesopores (Fig. S3). This result, forming porous structure of crystalline Fe/MnO@C-amorphous carbon composition, is coordinated with that of TEM and XRD. The large specific surface area and porous structure benefit the improvement of impedance matching behavior and microwave attenuation [38–44]. The surface areas and pores is conducive to impedance of fillers and boosts microwave penetrating into materials, which is a competent way to broaden absorption bandwidth. Meanwhile, abundant pores in filler can generate multiply scattering of microwave because of extending the transmission path of incident electromagnetic wave. In addition, the specific carbon content and iron content are ascertained by thermogravimetric (TG) analysis and X-ray fluorescence spectroscopy in composites as shown in Fig. S4.

From the TEM images and XRD result aforementioned, graphitization degree of FMCA composite is low and bonding state of carbon atoms is observed differently. Meanwhile, Raman spectroscopy is used to identify the degree of graphitization in carbonaceous materials. In Fig. 3b, the prepared amorphous carbon embedded with dispersed Fe/MnO@C nanocapsules present two prominent peaks at around 1580 (D band) and 1348 cm^−1^ (G band). G band denotes the vibration of *sp*^2^ hybridization, and D band denotes *sp*^3^ defects and disorder, respectively [45]. In general, the bigger *I*_D_/*I*_G_ value (intensity ratio of D to G band) is, the higher degree of disorder signifies. The calculated values of *I*_D_/*I*_G_ for FMCA-1, FMCA-2, FMCA-3, and FMCA-4 are 0.86, 0.87, 0.97, and 0.81, respectively. Therefore, the *I*_D_/*I*_G_ FMCA-1, FMCA-2, FMCA-3, and FMCA-4 exhibits uptrend except FMCA-4, which ascribes to the increase in additive amount of MnO and decrease in carbon amount. When the dosage of MnO is gradually increased, relative content of amorphous carbon is further consolidated. And the content of MnO testifies uptrend as the intensity of peak at 621 cm^−1^ increasing. Additionally, 2D peak appears at 2689 cm^−1^ indicating several graphene layers in FMCA composition, which is in agreement with high resolution TEM images (Fig. 2). Note that, *I*_D_/*I*_G_ for FMCA-4 is obvious extraordinary from other samples. This is attributed to the low amount of carbon materials. The surface chemical composition and valence states are further performed by XPS.

As shown in Fig. 3c, the survey scan taken from FMCA-3 confirms the presence of Fe, Mn, O, and C elements. From Fig. 3d, the peaks at 284.5, 286.1, and 289.0 eV of C 1s can be assigned to C–C/C=C, C=O, and C–OH bonds, respectively [46]. In Fig. 3e, the O 1s spectrum displays three peaks at 532.3, 530.8, and 529.2 eV, which ascribes to residual structure water (H–O–H), hydrated manganese oxides (Mn–O–H), and anhydrous manganese oxides (Mn–O–Mn), respectively. From Fig. 3f, the Fe 2*p* spectrum exhibits two peaks at 684.0 and 686.1 eV, which correspond to the Fe 2*p*_3/2_ and Fe 2*p*_1/2_, respectively. Thus, the analysis results of XPS reveal the synthesis of metallic Fe in the sample. As shown in Fig. 3g, the fitted Mn 2*p* spectra exhibit Mn^2+^, consistent with the results obtained from XRD.

**Fig. 4:**
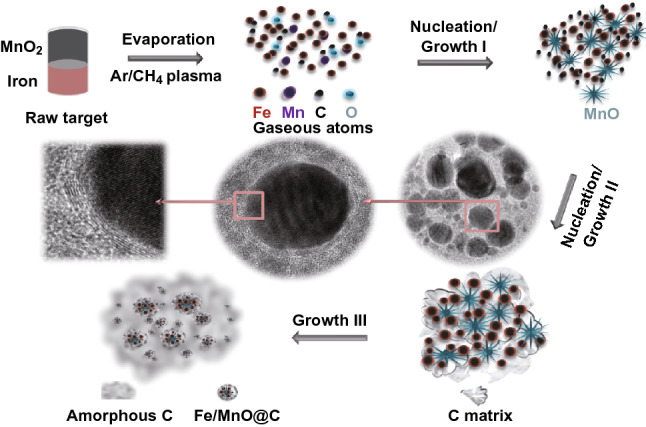
Schematic diagram for formation of FMCA composition

In basics of structural characterization, the formation process for FMCA composition is well comprehended as shown in Fig. 4. There are three stages in forming process, i.e., the evaporation, nucleation/growth, and catalyzed graphitization. In the first stage, the raw materials of bulk Fe and MnO_2_, gaseous CH_4_ are completely co-evaporated by high-energy arc-plasma into gaseous atoms. In the second stage, the evaporated atoms of raw species will orderly undergo nucleation and growth. It depends on the melting points of MnO (1650 °C) and Fe (1600 °C). The MnO and Fe seeds will nucleate once achieving melting point. At the same time, the diffusion of Fe and MnO is companied with nucleation because of a very similar melting point. The MnO nanoparticle restricts the Fe cluster extensive grown and the particles are in nanometer-scale. The prepared Fe/MnO nanocrystals exhibit their sphere-like morphologies and the amorphous carbon matrix make them protected from oxidization. Supersaturated carbon in the Fe/MnO lattice undergoes fast quenching during escaping out of the central arc district to the water-cooled chamber wall. The saturated carbon atoms separate into the carbon matrix where considerable stress is produced in-plane with the carbon layers. At the last stage, amorphous carbon adjacent to Fe/MnO nanocrystal is converted to crystalline graphite due to catalysis of iron. On the one hand, the wrapped amorphous carbon matrix and the crystalline graphite are involved in the protection of Fe nanocrystal against oxidative damage. The normal stress concentrates at the largest convex curvature of the ellipsoid-like nanoparticles and creates serious deformation of the carbon layers. On the other hand, Fe nanoparticles are confined in the crystalline graphite and disperse uniformly with MnO. The novel configuration can suppress the agglomeration of magnetic particles and consequent skin effect, and contribute good chemical homogeneity and sufficient interfaces between Fe/MnO nanoparticles and C. Besides, the addition of MnO avoids the formation of Fe_3_C in conventional iron–carbon compounds.

### Static Magnetization

**Fig. 5:**
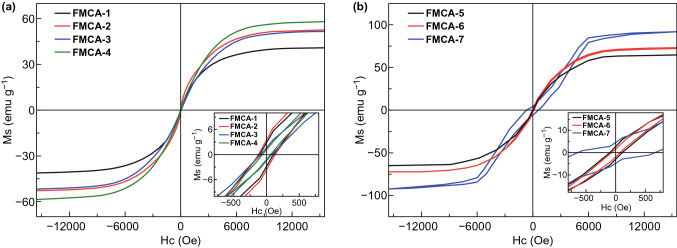
**a**, **b** Magnetization hysteresis loops at 300 K and insert is the corresponding magnification around the origin

The electromagnetic wave absorption property is closely related to the magnetic performance. Theoretically, magnetic loss ability ($\mu^{\prime \prime }$) is tightly related to magnetization value. As explained by the equation: $\mu^{\prime \prime } = \left( {M/H} \right) \sin \sigma$, where *M* represents the magnetization, *H* is the external magnetic field, σ is the phase lag angle of magnetization behind external magnetic field. According to the equation, a higher magnetization value results in improvement of magnetic loss ability ($\mu^{\prime \prime }$) value [47]. Figure 5a depicts the magnetic hysteresis loops of FMCA-1, FMCA-2, FMCA-3, and FMCA-4. Obviously, the samples present typical ferromagnetic hysteresis behavior with saturation magnetization (*M*_s_) being 41.1, 52.7, 51.8, and 58.3 emu g^−1^, respectively, benefiting for the magnetic loss [48]. As is well known, the magnetic properties derive from coupling between electron spin and its orbital angular momentum [49]. Nanoparticles always show superparamagnetic properties due to magneto crystalline anisotropy, which is different from bulk materials. The magnetic particles in Fe/MnO nanocrystals are deemed as single-domain particles with critical diameter (Dc) close to 20 nm. The magnetic anisotropy energy is responsible for holding the magnetic moments along a certain direction, which is expressed as $E\left( \theta \right) = K_{\text{eff}} V\sin^{2} \theta$, where $V$ donates volume of the particle, $K_{\text{eff}}$ is the anisotropy constant, and $\theta$ is the angle between the magnetization and the easy axis. The magnetization with two energetically easy directions is separated by the energy of barrier $K_{\text{eff}} V$. If the particle volume $V$ is extremely small, the thermal energy ($K_{\text{B}} T$) is sufficient to overcome the anisotropy barrier ($K_{\text{eff}} V$) of the orientation of a well-isolated single-domain particle. When $K_{\text{B}} T > K_{\text{eff}} V$, a single nanoparticle behaves like a giant paramagnet and is superparamagnetic. This can be the reason that the saturation magnetization of the samples presents uptrend even through the Fe content decrease. Furthermore, metal nanoparticles are easily oxidized, and value of *Ms* is high with minimizing ratio of oxidation. Meanwhile, the nanoparticle size is controlled to avoid a reduction of *Ms* because of oxides and surface disorder [50]. Herein, the above problems can easily be overcome in this work. Since the crystalline Fe/MnO@C-amorphous carbon composition is subtly constructed to control the oxidation and the particle size xudong.wang@wisc.edu (Xudong Wang); caozq@dlut.edu.cn (Zhiqiang Cao); ymao@zzu.edu.cn (Yanchao Mao) while taking the advantage of different existing forms of graphite, which is easy to scale up and confine the sacrifices of magnetic loss. Moreover, by adjusting the Fe mass ratios to 60%, a strong magnetization can be achieved as shown in Fig. 5b. The rationally designed FMCA composites reveal an excellent magnetic performance, which opens up a new path to restrict sacrifices of magnetic loss.

### Electromagnetic Parameters and Microwave Absorption Performance

**Fig. 6:**
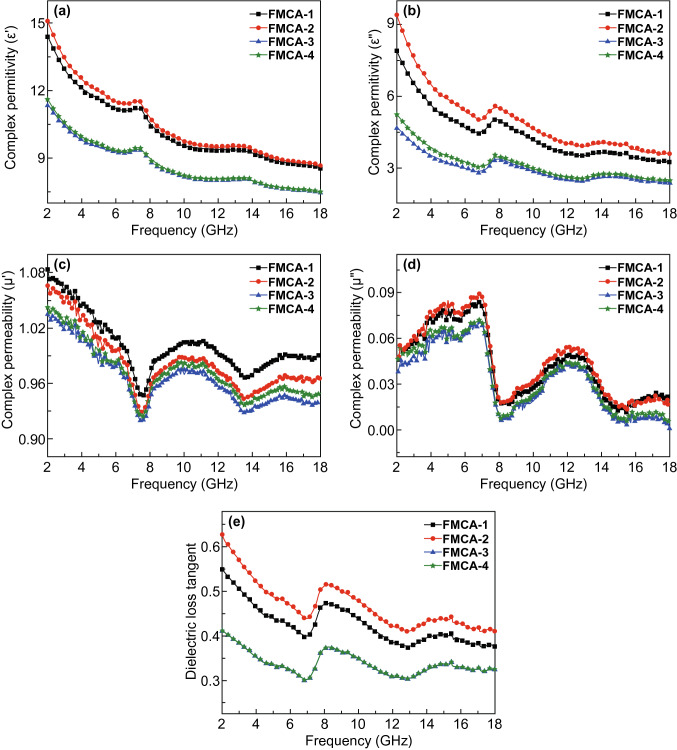
Electromagnetic parameters of FMCA: **a** real part $\left( {\varepsilon^{\prime}} \right)$, **b** imaginary part ($\varepsilon^{\prime\prime}$) of permittivity, **c** real part ($\mu^{\prime}$), **d** imaginary part $\left( {\mu^{\prime \prime }} \right)$ of permeability, **e** dielectric loss tangent

The electromagnetic wave absorption performance mainly depends on complex permittivity ($\varepsilon_{r} = \varepsilon^{\prime} - j\varepsilon^{\prime\prime}$) and permeability ($\mu_{r} = \mu^{\prime} - j\mu^{\prime\prime}$) [51, 52]. The complex permittivity for the Fe@C composition is shown in Fig. S5. Both $\varepsilon^{\prime}$ and $\varepsilon^{\prime\prime}$ decrease rapidly with adding MnO_2_ during the reaction as shown in Fig. 6a, b. Figure 6c, d displays the complex permeability. The $\mu^{\prime}$ value exhibits downtrend after the addition of MnO. Resonance peaks appear in the $\mu - f$ curves (Fig. 6c, d). Large values of $\varepsilon^{\prime}$ and $\varepsilon^{\prime\prime}$ endow strong attenuation capability (Fig. S6), which may lead to a poor impedance matching. The dielectric loss tangents ($\tan \delta_{\text{e}} = \varepsilon^{\prime\prime}/\varepsilon^{\prime}$) further exhibit that dielectric loss ability of theses composites. The order of dielectric loss ability is same as that of complex permittivity (Fig. 6e). The dielectric loss tangent also decreases with addition of MnO. Compared with dielectric loss tangent, the value of magnetic loss tangent is small for FMCA samples (Fig. S7a). This is mainly put down to the introduction of nonmagnetic MnO and carbon matrix.

The dielectric loss, magnetic loss, and impedance matching determine collectively electromagnetic wave absorption performance [53]. The magnetic loss mainly comes from domain-well resonance, hysteresis loss, eddy current effect, natural and exchange resonance [54]. The domain-wall resonance is excluded from the GHz range. And the hysteresis loss is negligible in weak applied field [55]. When magnetic loss only originates from eddy current effect, $C_{0} = \mu^{\prime \prime }\left( {\mu^{\prime}} \right)^{ - 2} f^{ - 1}$ should tend to be a straight line. *C*_0_ is fluctuant over 2–18 GHz (Fig. S7b), it indicates that the natural resonance has also been devoted to the magnetic loss. The peaks around 7.3 GHz are observed in Fig. 6c, which attributes to the natural resonances of the FMCA-1, FMCA-2, FMCA-3, and FMCA-4. $f_{\text{r}} = 2\gamma K_{\text{eff}} /M_{\text{s}}$ expresses frequency of natural resonance, where $K_{\text{eff}}$ donates the effective anisotropy constant, $\gamma$ the gyromagnetic ratio, and $M_{\text{s}}$ the saturation magnetization [56]. The effective anisotropy constant relies on the size of particles and surface effect, given by $K_{\text{eff}} = K_{\text{v}} + 6K_{\text{s}} /R$, where $K_{\text{v}}$ represents the volume anisotropy constant, $K_{\text{s}}$ the surface anisotropy constant, and $R$ the radius of the nanoparticles [57]. $K_{\text{eff}}$ is enhanced with small size of the Fe/MnO@C nanocapsule due to the confinement effect. $M_{\text{s }}$ of the crystalline FMCA composition decreases as the introduction of nonmagnetic MnO compared to that of Fe@C composition. However, value of $M_{s }$ presents uptrend even through the decrease content of magnetic Fe. Hence, $f_{\text{r}}$ appears in higher frequency of 7.3 GHz. Resonance peak around 13.6 GHz ascribes to the exchange resonance since it has been well-documented to locate at high frequency [58, 59].

**Fig. 7:**
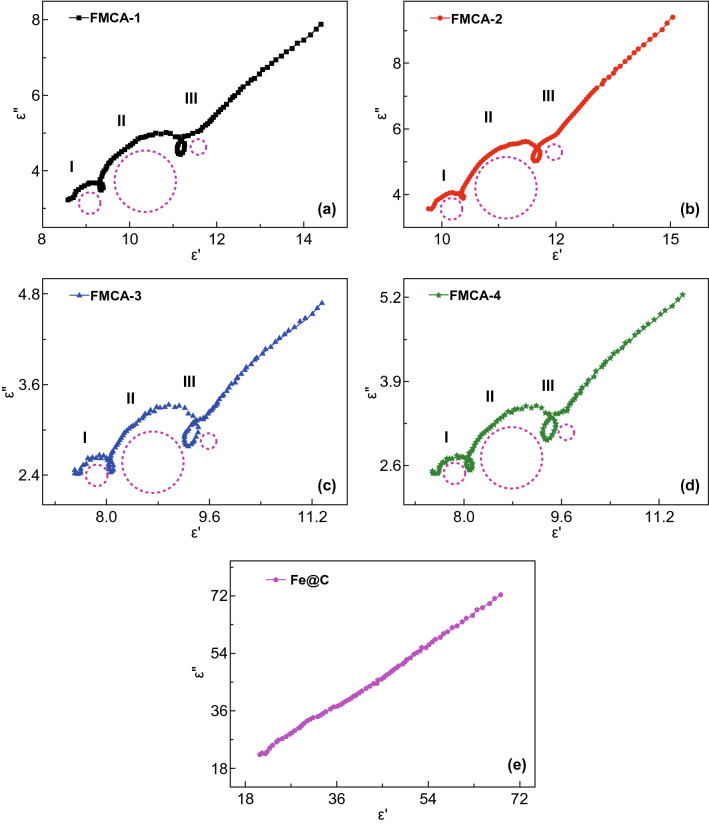
Cole–Cole curves of **a** FMCA-1, **b** FMCA-2, **c** FMCA-3, **d** FMCA-4, and **e** Fe@C

Generally, dielectric loss primarily derives from polarization and conductivity loss. And polarization loss includes dipolar polarization, interfacial polarization, ionic polarization, and electronic polarization. Ionic polarization and electronic polarization are excluded in microwave range because they appear at high frequency of 10^3^–10^6^ GHz. Thus, the main dielectric attenuation mechanism is dipolar polarization and interfacial polarization. The dipolar polarization appears at molecule with obvious dipole moment. The interfacial polarization usually generates in a heterogeneous structure due to the accumulation and uneven distribution of space charges. The polarization process is investigated via Cole–Cole curves based on $\left( {\varepsilon^{\prime} - \left( {\varepsilon_{\text{s}} + \varepsilon_{\infty } } \right)/2} \right)^{2} + \left( {\varepsilon^{\prime\prime}} \right)^{2} = \left( {\left( {\varepsilon_{\text{s}} - \varepsilon_{\infty } )/2} \right)^{2} } \right)$ [60]. The excessive dielectric loss of Fe@C composition leads to mismatched impedance due to formation of the conductive Fe and graphite network. For respective Cole–Cole curves of FMCA-1, FMCA-2, FMCA-3, and FMCA-4 (Fig. 7), three semicircles are observed obviously, manifesting significant polarization in composites. The heterogeneous interface between Fe/MnO nanocrystals and crystalline graphite, crystalline graphite, and amorphous carbon endows multiple dielectric relaxation processes. But simultaneously, the Cole–Cole curves show long tail and semicircles distort somewhat. This indicates that the conductive loss also dominates in the dielectric loss. Introduction of MnO nanoparticles rationally can inhibit iron agglomeration and decrease conductivity, forming the homo-disperse of Fe and MnO, and generate interface between carbon matrix and Fe/MnO nanocrystal. For comparison, Cole–Cole curves plot is line and no semicircles can be observed of the Fe@C composition (Fig. 7e). This reveals conduction loss dominates for dielectric loss for Fe@C composition. Thus, the FMCA composition interrupts the Fe and carbon conductive network. The dielectric loss decreases for FMCA-1, FMCA-2, FMCA-3, and FMCA-4 based on the free electron theory [38, 61].

**Fig. 8:**
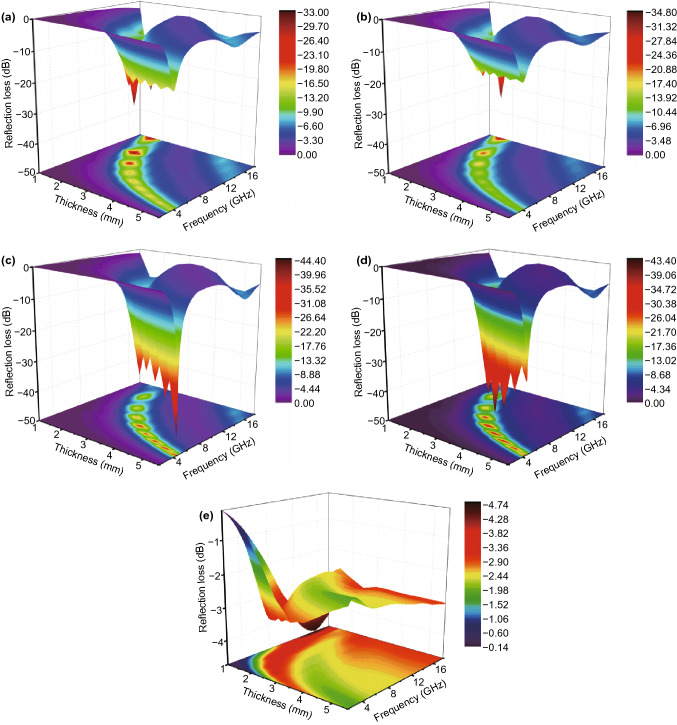
Three-dimensional reflection loss maps of **a** FMCA-1, **b** FMCA-2, **c** FMCA-3, **d** FMCA-4, and **e** Fe@C over 2–18 GHz

RL of FMCA composition is calculated in basic of the complex permittivity and complex permeability (Fig. 8a–d). Improved performance is achieved after addition of MnO. The minimum reflection loss for FMCA-1 exhibits − 37.5 dB at 12.5 GHz together with an effective absorbing bandwidth of 4.8 GHz at a small absorber thickness of 2 mm (Fig. 8a). The FMCA-2 shows absorption property with minimum reflection loss of − 22.3 dB and an effective absorption bandwidth of 5.1 dB with thin thickness of 2 mm (Fig. 8b). With increased MnO_2_ content, the minimum reflection loss reaches − 45 dB with thickness of 5.5 mm for FMCA-3 and 4 mm for FMCA-4 (Fig. 8c, d). And the effective absorption bandwidth achieves 5.0 dB at thickness of 2 mm. The minimum reflection loss value for Fe@C composition is only − 5.6 dB due to the large dielectric loss (Fig. 8e).

**Fig. 9:**
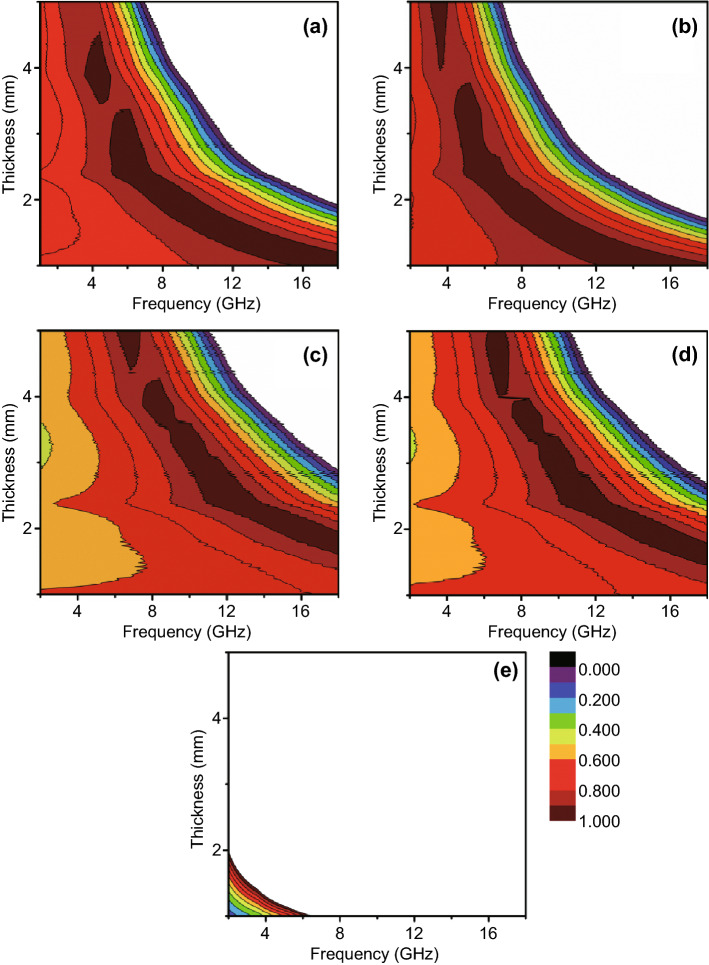
Impedance matching degree dependence of frequency of **a** FMCA-1, **b** FMCA-2, **c** FMCA-3, **d** FMCA-4, and **e** Fe@C

Visibly, such excellent absorbing properties for FMCA-3 primarily ascribes to well impedance matching degree ∆. In general, large integration area of ∆ values below 1.0 of FMCA-3 demonstrates the better impedance matching in Fig. 9c. The reflection of electromagnetic wave is avoided at front surface of the absorbers [62, 63]. $\alpha$ and ∆ can be realized optimum balance in FMCA-3. Moreover, the FMCA compositions possess excellent impedance matching compared with that of Fe@C composition (Fig. 9e), revealing the microwave absorption capabilities of FMCA compositions can be controllable by regulating the addition of MnO_2_.

**Fig. 10:**
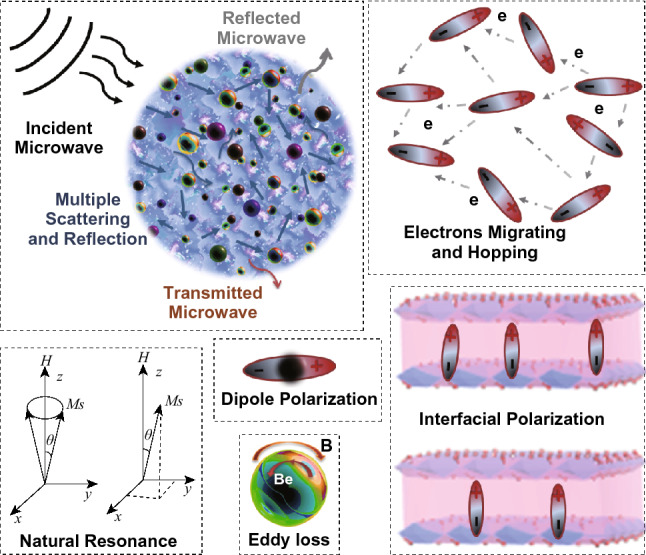
Schematic illustrations for electromagnetic wave absorbing mechanism

Overall, the outstanding microwave absorption performance of FMCA composites ascribes to the synergistic effect of dielectric loss and magnetic loss, derived from the interfacial polarization and moderate conductive loss of Fe and carbon matrix, along well with natural resonance and exchange resonance (Fig. 10). Firstly, dispersed MnO nanocrystals avoid agglomeration of Fe particles with small size, as shown in Fig. 2a, c. And the introduction of MnO decreases the degree of graphitization and fascinates the formation of amorphous carbon matrix. Then, the heterogeneous interfaces between Fe/MnO/crystalline graphite/amorphous carbon matrix give rise to interfacial polarization and large amounts of defects formed, acting as polarized centers which combine to boost electromagnetic absorption performance. Secondly, uniformly dispersed MnO suppresses the stacking of graphene and agglomeration of magnetic particles, consequent restraining skin effect. Meanwhile, the impedance matching is well regulated. The well impedance matching guarantees electromagnetic wave entering the absorber. Inside the absorber, the multiple scattering and reflecting of microwave increase the propagation paths. This is benefit for the attenuation of microwave energy. Third, nanoscale Fe/MnO nanocrystals in carbon matrix intensely response to broad-band microwave based on their induced currents. The electromagnetic energy can be converted to the thermal energy quickly [64].

## Conclusions

The crystalline core–shell Fe/MnO@C nanocapsule inlaid in porous amorphous carbon is synthesized by modified arc-discharge method. The formed well-distributed Fe/MnO nanocrystals imped Fe aggregation and interruption of graphite conductive network due to introduction MnO, resulting in tunable dielectric loss of the heterostructure. An increase in MnO brings about natural resonance in a specific frequency, multi-reflection at the interface of Fe and carbon, dielectric polarization, electron-jumping multi-phase envelop structure and size effect. Consequently, good impedance matching and strong attenuation capacity can be obtained because of the synergistic effect in the magnetic and dielectric loss. The optimal reflection loss achieves − 45 dB for FMCA-3, and effective absorption bandwidth achieves 5.0 dB with 2 mm thickness. This study not only provides insights on the design of advanced microwave absorbing materials with low density and broadband absorption, but also paves a versatile way for the large-scale synthesis of porous composites for extended applications.

## Electronic supplementary material

Below is the link to the electronic supplementary material.

Supplementary material 1 (PDF 696 kb)
