# Supplementary material for: Microwave Absorption of Crystalline Fe/MnO@C Nanocapsules Embedded in Amorphous Carbon
Source: Nanomicro Lett. 2020 Feb 18;12:57. doi: 10.1007/s40820-020-0388-4 (PMC7770782; doi:10.1007/s40820-020-0388-4)
Supplement: Supplementary file 1 — Supplementary material 1 (PDF 696 kb) [file 40820_2020_388_MOESM1_ESM.pdf]

Supporting Information for

Microwave Absorbents of Crystalline Fe/MnO@C Nanocapsules Embedded in Amorphous Carbon

Gaihua He[1], Yuping Duan[1,*], Huifang Pang[1]

[1]Key Laboratory of Solidification Control and Digital Preparation Technology (Liaoning Province), School of Materials Science and Engineering, Dalian University of Technology, Dalian 116085, People's Republic of China

*Corresponding author. E-mail: duanyp@dlut.edu.cn (Yuping Duan)

Supplementary Figures

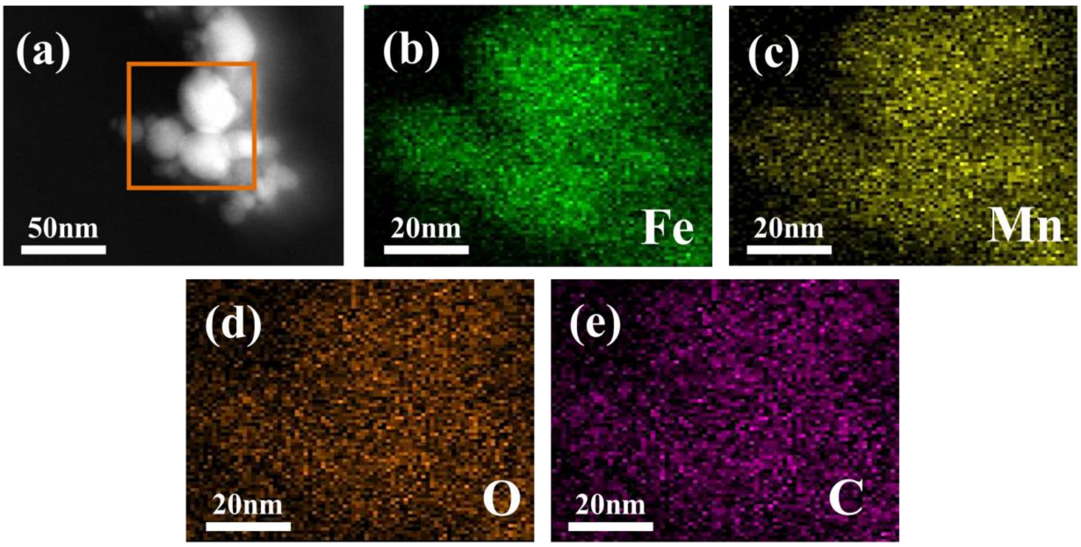

Fig. S1 Electron energy loss spectroscopy (EELS) elemental mapping of crystalline Fe/MnO_2@C-amorphous carbon composition

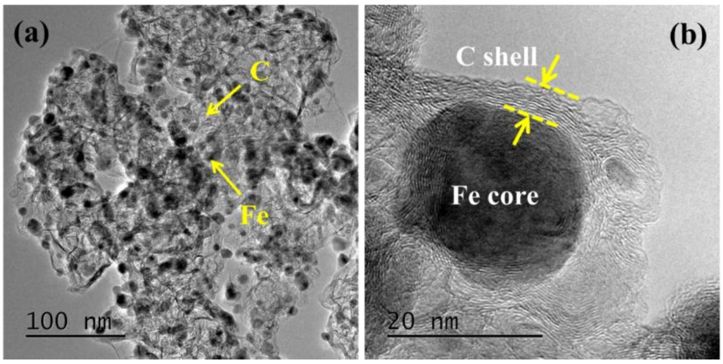

Fig. S2 TEM images and high resolution TEM images of Fe@C samples

The specific surface area is 51.01 m^2 g^{-1} as shown in Fig. S3a, larger than that of

Fe@C composition (Fig. S3c). Barrett-Joyner-Halenda (BJH) result demonstrates that the average pore diameter of FMCA-3 is approximate 3 nm (Fig. S3b). The tolal pore volume is 0.0794 cm^3/g, which is larger than that of Fe@C composition (Fig. S3d).

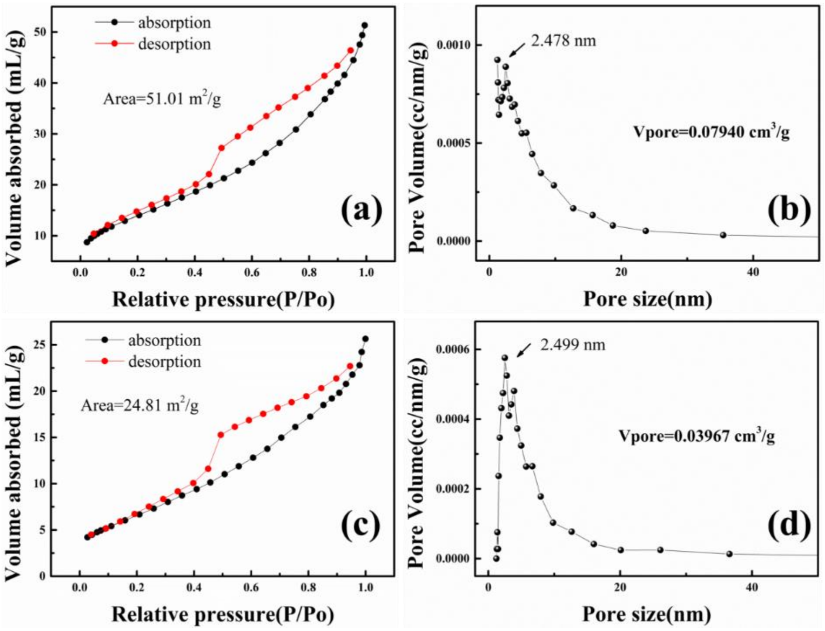

Fig. S3 N_2 adsorption desorption isotherms (**a**) and pore sized distribution (**b**) for sample FMCA-1, N_2 adsorption-desorption isotherms (**c**) and pore sized distribution (**d**) for sample Fe@C

TG analysis is widely utilized to determine the specific carbon content in carbon-based composites. As shown in Fig. S4a, FMCA displays a very weak weight loss in the temperature range of 100-150 °C owing to the removal of trace absorbed water and surface groups. With further increasing the temperature, the oxidation of Fe nanoparticles and combustion of carbon species will account for a sharp weight adding until the residual mass is unchanged. Obviously, the combustion of carbon species is companied with the oxidation of Fe nanoparticles. The carbon content cannot be estimated only according the TG analysis. Therefore, X-ray fluorescence spectroscopy is further taken to measure the Fe and carbon content. The result is shown in Fig. S4b.

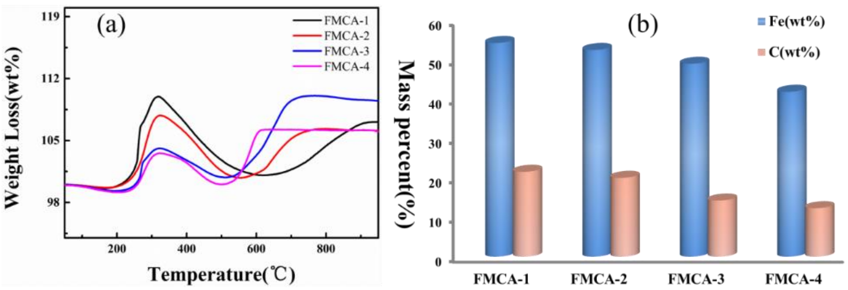

Fig. S4 TG curves under air atmosphere (**a**), Fe and carbon content of FMCA composites (**b**)

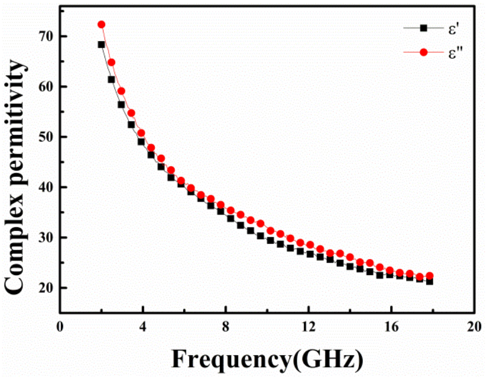

Fig. S5 Complex permittivity for the Fe@C composition

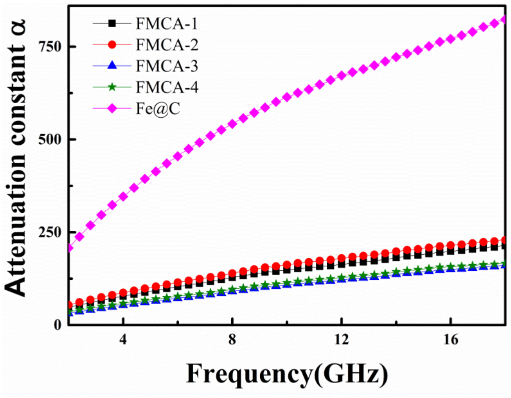

Fig. S6 Relationship between attenuation coefficient α and frequency

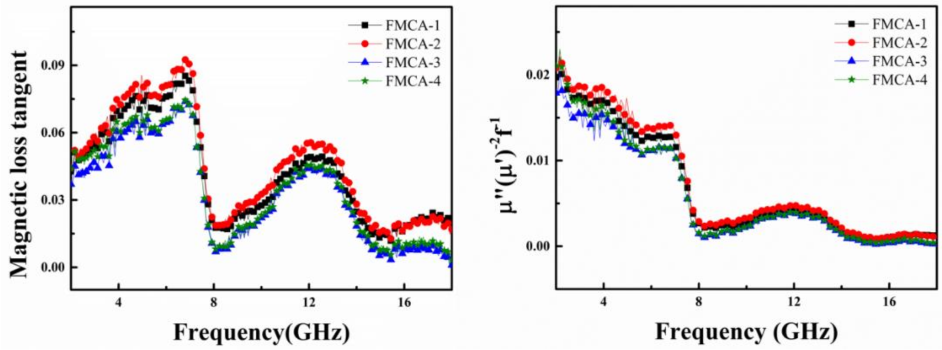

Fig. S7 a Magnetic loss tangent and **b** frequency dependent C_0 ($C_0 = \mu''(\mu')^{-2}f^{-1}$) values of FMCA composition in the frequency range 2-18 GHz (if the magnetic loss originates from the eddy current effect, C_0 should tend to be a straight line. if C_0 is fluctuant, it can be explained that the natural resonance also contributes the magnetic loss)

The strong magnetization can be achieved shown in Fig. 5b, which is adjusted by Fe mass ratios. Fe mass ratios of FMCA-5, FMCA-6, and FMCA-7 are 55%, 57%, and 60%, respectively. The saturation magnetization value of FMCA-7 achieves 92.30 emu/g, which is even bigger than that of Fe@C composition (78.57 emu/g). Thus, the rationally designed FMCA composites endow an excellent magnetic performance, which opens up a new path to restrict sacrifices of magnetic loss.
